# *Schmidtea mediterranea* as a Model Organism to Study the Molecular Background of Human Motile Ciliopathies

**DOI:** 10.3390/ijms24054472

**Published:** 2023-02-24

**Authors:** Alicja Rabiasz, Ewa Ziętkiewicz

**Affiliations:** Institute of Human Genetics Polish Academy of Sciences, 32 Strzeszyńska Street, 60-479 Poznań, Poland

**Keywords:** candidate genes, flatworms, motile cilia, planarians, primary ciliary dyskinesia (PCD), RNA interference (RNAi)

## Abstract

Cilia and flagella are evolutionarily conserved organelles that form protrusions on the surface of many growth-arrested or differentiated eukaryotic cells. Due to the structural and functional differences, cilia can be roughly classified as motile and non-motile (primary). Genetically determined dysfunction of motile cilia is the basis of primary ciliary dyskinesia (PCD), a heterogeneous ciliopathy affecting respiratory airways, fertility, and laterality. In the face of the still incomplete knowledge of PCD genetics and phenotype-genotype relations in PCD and the spectrum of PCD-like diseases, a continuous search for new causative genes is required. The use of model organisms has been a great part of the advances in understanding molecular mechanisms and the genetic basis of human diseases; the PCD spectrum is not different in this respect. The planarian model (*Schmidtea mediterranea*) has been intensely used to study regeneration processes, and—in the context of cilia—their evolution, assembly, and role in cell signaling. However, relatively little attention has been paid to the use of this simple and accessible model for studying the genetics of PCD and related diseases. The recent rapid development of the available planarian databases with detailed genomic and functional annotations prompted us to review the potential of the *S. mediterranea* model for studying human motile ciliopathies.

## 1. Introduction–Cilia and Ciliopathies

Cilia and flagella are evolutionarily conserved organelles that form protrusions on the surface of many growth-arrested or differentiated eukaryotic cells, from simple unicellular organisms such as *Chlamydomonas* to specialized cells in higher animals—fish and mammals [1,2,3]. The cilium is anchored at the cell membrane via the basal body, which extends into the axoneme, the main part of the cilium protruding outside the cell; the transition zone between the basal body and axoneme has a function of ciliary gating [4]. Due to structural and functional differences, cilia can be roughly classified as motile and non-motile (also called primary or sensory), although their detailed classification is much more complicated [5,6].

**Motile cilia** are ancient organelles that were already present in the Last Eukaryotic Common Ancestor, LECA [2]. Their main function is associated with their ability to move; they also perform some sensory functions [5,7,8]. In unicellular organisms, such as *Chlamydomonas*, *Paramecium*, or *Tetrahymena*, cilia or flagella (structurally related to motile cilia, but much longer) are responsible for the movement of cells. In higher organisms, motile cilia (5–10 um long) form a dense carpet (hundreds of organelles per cell) on the apical side of multiciliated cells (MCCs), and through their coordinated planar beating are responsible for the flow of fluids covering epithelium [9]. Single flagella are responsible for sperm motility [10], and single motile cilia in the mammalian embryonal node regulate the directional flow of signals required for the establishment of left-right patterning [11]. **Primary cilia** found as singular entities on the surface of almost every differentiated metazoan cell type [1], are not in focus in this review, but it should be mentioned that they evolved from motile cilia. Due to the difference in their structure, they are immotile [12], but they play an important role in the reception and transmission of signals involved in the regulation of cellular processes essential for development and the maintenance of tissue homeostasis. The main signaling pathways coordinated by primary cilia include those regulated by Hedgehog (HH), G-protein-coupled receptors (GPCR), wingless (WNT), receptor-tyrosine kinases (RTKs), and TGFβ/BMP receptors [12,13,14,15].

**Figure 1:**
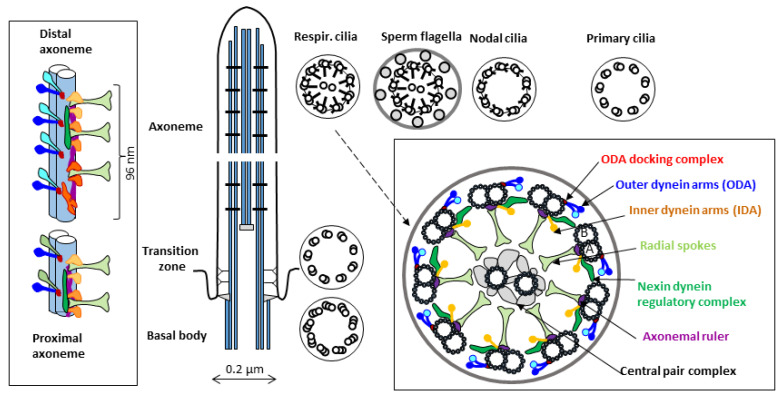
**Simplified schematic representation of the ultrastructure of a motile cilium** (longitudinal and cross sections detailing microtubule-associated elements). Cross-sections of the axoneme of different types of motile cilia (respiratory and nodal cilia, sperm flagellum) are compared with that of a primary cilium. The periodic longitudinal unit (organized in 96 nm repeats) consists of four ODAs (attached to A microtubules of the peripheral dublets through the ODA-docking complexes), three radial spokes, and a set of IDAs (consisting of different complexes identified using a cryoelectron tomography [16,19]). The length of this unit is determined by the molecular ruler proteins (in humans, CCDC39 and CCDC40, homologs of Chlamydomonas proteins FAP59 and FAP172 [20]). In mammalian motile cilia, ODAs differ in the proximal and distal parts of the axoneme (by the presence of DNAH11 and DNAH9 proteins, respectively); in sperm flagella, they are replaced by a single protein DNAH17 [21]. Several small elements attached to the A microtubule, not visible in classical TEM, have been identified in model organisms using cryoelectron tomography [22]; additional small proteins are docked at the luminal side of both A- and B-microtubules [23,24]. The central microtubules, connected by a multiprotein bridge, and surrounded by a set of projections, form the central pair complex [25].

In the axoneme of a typical motile cilium or flagellum (Figure 1), nine peripheral doublets of microtubules (A and B) surround two single central microtubules (C1 and C2). In the cross-section of the axoneme observed in the transmission electron microscope (TEM), this arrangement is known as the 9 × 2 + 2 pattern. Additional protein elements attached to the microtubules are distributed periodically along the axoneme length. Multiprotein complexes associated with the peripheral A microtubules form characteristic auxiliary structures: outer and inner dynein arms (ODA and IDA), ODA docking complexes, radial spokes (RS), and nexin-dynein regulatory complexes (N-DRC). Several detailed reviews describing the axonemal structure components have been published in recent years, e.g., [16,17,18].

Cilia movement is powered by ODAs and IDAs, which act as ATP-dependent molecular motors. They move in synchrony along the B microtubule of the adjacent doublet. The resulting mutual sliding of the peripheral doublets is restricted by the N-DRCs, which connect neighboring doublets; the net result is a bend of the cilium [26]. Radial spokes, attached to the peripheral A microtubules and transiently contacting the projections of the central microtubules, stabilize the structure and functionally connect the central apparatus to the dynein arms [27]. The cooperation of all these ultrastructural elements results in a coordinated planar beating of motile cilia, with a fast power stroke and a slower recovery stroke occurring in the same plane; for the review see, e.g., [18].

The ciliogenesis of motile cilia is a multi-step process, starting from the cell cycle exit and involving many signaling factors; it has been the subject of many excellent reviews, e.g., [28]. The inhibition of the NOTCH1 signal and activation of the MCIDAS-dependent pathway initiates the biogenesis process specific to MCCs [29]. In MCCs, the MCIDAS pathway orchestrates massive centriole amplification and their docking in the apical cell membrane (through the activation of cyclin O) [30]. The expression of the FOXJ1 transcription factor switches on the synthesis of proteins directly involved in the formation of motile cilia ultrastructure [31]. Cilia elongation and maintenance are possible thanks to the presence of a dedicated protein shuttle system, named intraflagellar transport (IFT), which involves the transport of molecules from the cell body through the basal body, transition zone to the tip of cilia and back [32,33]. Some of the multiprotein ciliary elements of motile cilia (e.g., dynein arms, ODA docking complexes) are preassembled in the cytoplasm in a process that requires the presence of proteins, which are physically not a part of the axonemal ultrastructure [34,35].

Genetically determined dysfunction of cilia is the cause of a large group of diseases, collectively referred to as ciliopathies. With the expanding knowledge of cilia biology and genetics, their number is now estimated as at least 35 [36]. The majority of ciliopathies are caused by the dysfunction of primary/sensory cilia [37]. Consistent with the presence of primary cilia on almost all cell types and their function in basic cellular pathways, their dysfunction affects multiple systems, including kidneys, brain, heart, skeleton, and eyes (e.g., polycystic kidney disease, PKD; nephronophthisis, NPHP; Bardet-Biedl syndrome, BBS; Joubert syndrome, JBTS; Meckel syndrome, MKS; Senior-Locken syndrome, SLSN; retinitis pigmentosa, RP); for the reviews see [36,37,38]. In this review, however, we focus on the diseases caused by the genetically determined defects of the structure and/or function of **motile** cilia.

### 1.1. Primary Ciliary Dyskinesia

Primary ciliary dyskinesia, PCD (OMIM244400; population frequency of 1:10,000 to 1:20,000), is the flagship ciliopathy resulting from the dysfuntion of motile cilia [34,35,39,40,41,42,43]. It has to be emphasized that the word “primary” in the name of the disease refers to the fact that PCD is a genetically-based condition, as opposed to a “secondary” ciliary dyskinesia, where the dysfunction of motile cilia is caused by environmental factors.

Typical clinical symptoms of PCD reflect the role of motile cilia in different parts of the human body. Defects of cilia on the apical surface of the epithelial cells lining the respiratory tract impair mucociliary clearance; as a result, PCD patients suffer from recurrent respiratory airway infections leading to chronic bronchopulmonary disease, recurrent sinusitis, rhinitis, otitis media, and bronchiectasis. The immotility of sperm flagella is a cause of male infertility, and the immotility of cilia on the epithelial cells lining fallopian tubes reduces fertility in females. Dysfunction of cilia present on the ependymal cells in the brain, which are responsible for cerebrospinal fluid flow, can lead to hydrocephalus. Finally, the defects of single motile cilia in the embryonic node impair the flow of morphogens and body patterning (situs), resulting in the randomization of body organ symmetry (*situs inversus totalis* in about 50% of PCD patients).

Due to the largely nonspecific clinical symptoms of PCD and insufficient diagnostic methods, PCD patients are often diagnosed late. The range and severity of PCD symptoms depend on the mutated gene, reflecting different impacts of the dysfunctional protein on the ciliary structure and/or function [34,35,42,43,44,45].

Theoretically, the diagnostic problems can be overcome by applying genetic tests with high efficiency of mutation detection. However, the genetic basis of PCD is highly heterogeneous, reflecting a large number of proteins involved in the structure and function of motile cilia [46,47]. The inheritance of PCD in most families is autosomal recessive; the X-linked or autosomal dominant inheritance is rare. To date, ~50 genes have been reported to be involved in PCD pathogenesis (Table 1), and the involvement of more is still under investigation [34,35,39,40,42,44] (Table 2). The role of these genes in the axonemal structure, ciliary function, or in the biogenesis of motile cilia has been confirmed in animal models using a range of approaches, including analysis of mutant organisms, knockout of the gene in question by targeted mutation or gene knockdown using double-stranded RNA (dsRNA) or morpholinos.

Pathogenic variants in many of the PCD genes result in obvious ultrastructural and/or functional defects of cilia, which can be readily visualized using transmission electron microscopy (TEM) or analysis of the ciliary beat [45,169,170]. The most frequent ultrastructural defects include the absence or shortening of ODA or both ODA and IDA, disorganization of microtubule arrangement, scarcity, or a complete lack of cilia. The defects of the ciliary beat can manifest as immotility, flickering, slow beating, or disturbed pattern of beating [169,170]. On the other hand, mutations identified in some of the genes, e.g., encoding central pair complex proteins, N-DRC proteins, or some of the dynein arm elements, have no clear effect on the axonemal structure or on the cilia beat frequency [102,119,123,139,147,171].

Importantly, even the use of high-throughput genome sequencing fails to detect mutations in known PCD genes in ~1/3 of the patients [34,35]. This may be due to the presence of unknown pathogenic variants, lying outside the most commonly studied coding sequences or impossible to detect with copy number insensitive techniques, as well as the presence of pathogenic mutations in yet-unidentified PCD genes. Moreover, the association of some candidate genes with PCD pathogenesis remains a matter of debate, especially when mutations have been described in single families. In addition, there is an expanding list of genes from the so-called PCD spectrum [172], in which mutations in cilia-related genes are associated with an atypical clinical picture of the disease, with a syndromic presentation [51,52,54,55,173] or without (or very mild) respiratory symptoms [10,102,174,175,176]. Classification and curation of gene-disease relationships involving PCD and related motile cilia disorders are currently the focus of the Motile Ciliopathy Gene Curation Expert Panel, a part of the ClinicalGenome consortium [https://www.clinicalgenome.org/ (accessed on 20 February 2023)] [177].

The overall result of the heterogeneity of heritable motile ciliopathies is that—to better characterize the molecular/genetic basis of the unsolved cases of PCD and to differentiate them from the PCD-like spectrum diseases—the search for new candidate genes potentially involved in the pathogenesis is still needed.

### 1.2. The Validation of New Genes Underlying PCD and PCD-like Ciliopathies—The Role of Model Organisms

The easy access to NGS-based genetic screening of PCD patients increases the chances to reveal new candidate genes. Validation of their impact on motile cilia structure and function requires functional studies. The use of primary cell cultures in such studies requires obtaining respiratory epithelial biopsies from patients with pathogenic variants in candidate genes; the amount of the biological material obtained this way is limited, and often insufficient for detailed biochemical and molecular analyses. Another approach, silencing candidate genes in the in vitro culture of healthy human respiratory epithelium (HRE), is not in a routine laboratory method (reviewed in [178]). Primary HRE cells have limited ability to proliferate in culture. While the recent development of conditionally reprogrammed HRE cell cultures increased the proliferative lifespan of these cells and their ability to differentiate, this model is very demanding and still not sufficiently robust and replicable [178]. This is especially important in functional studies, where genome modification is required to overexpress or silence specific candidate genes.

Thanks to the high level of evolutionary conservation of motile cilia [3], a variety of model organisms have been successfully used for studying cilia biology. The same approach, which has allowed explaining the molecular basis of cilia assembly, structure, and function across Eukaryotic species, is widely used as a tool in the functional analysis of candidate genes underlying the pathogenesis of PCD and other cilia-related diseases.

For almost half of the causative genes identified during over twenty years of PCD research, their involvement in motile cilia function has been revealed by earlier (non-PCD) forward genetics studies in model organisms, performed to study cilia biology–the identity of proteins, their ultrastructural localization, interactions, and function. The loss-of-function variants in these genes, when found among patients, have been directly associated with PCD pathogenesis. For another half of PCD genes, with deleterious variants identified during the genetic screening of patients, their involvement in motile cilia dysfunction has been confirmed by follow-up reverse genetics studies, involving candidate gene silencing in model organisms.

The majority of all these studies had been based on a model of double-flagella unicellular alga, *Chlamydomonas reinhardtii*. *DNAI1*, the first gene identified as involved in the pathogenesis of PCD, has been earlier associated with the flagellar dysfunction caused by the mutation of *IC78*, *DNAI1* homolog in a double-flagella unicellular alga, *Ch. reinhardtii* [88]. Other models, which had supported the role of then-candidate PCD genes in motile cilia dysfunction, include unicellular organisms (*P. reinhardtii*, *Trypanosoma *brucei*, Tetrahymena thermophila*)*,* invertebrates (*Drosophila melanogaster, Schmidtea mediterranea*) or vertebrates (frog *Xenopus leavis,* fish *Danio rerio/*zebrafish, mouse, dog); reviewed in [179,180]. Among these model organisms considered in the context of PCD, relatively little attention has been put to the use of *S. mediterranea*.

## 2. *Schmidtea mediterranea*

*S. mediterranea* is a representative of freshwater planarians, free-living invertebrates from the phylum Platyhelminthes (flatworms). These animals belong to the group of organisms that have three germ layers (endoderm, mesoderm, and ectoderm), bilateral symmetry, and tissues with separate organs. The manner of reproduction of the freshwater planarians varies across the species and can be exclusively asexual (by transverse fission), seasonally sexual, and exclusively sexual (by the cross-fertilization of hermaphrodites) [181]. Planarians achieved popularity due to their great ability to regenerate after amputation or injury. In some cases, a full organism can be rebuilt after several days from 1/279 piece of a single worm, although the regenerative abilities of planarians are different across the species [182,183,184,185]. This regenerative potential makes planarians practically immortal and enables researchers to use them as efficient model organisms in a variety of studies.

There are several hundred species of planarians, but their use as animal models in molecular and genetic studies has been mostly limited to the Dugesiidae family (e.g., genera Dugesia, Girardia, Schmidtea), with the majority of research conducted using two species, *S. mediterranea* and *Dugesia japonica* [186]. *D. japonica* is favored for behavioral studies and toxicology screening, while *S. mediterranea* is popular and attractive for molecular experiments [187]. Two distinct strains of *S. mediterranea* exist in nature: a sexual strain (2 cm long) and an asexual strain (slightly shorter). Both are diploid, with four pairs of chromosomes (2n = 8); the asexual form results from a chromosome translocation between the sexual strain chromosomes 1 and 3 [181,188].

A planarian organism has a complex anatomy. The nervous system of flatworms is comprised of a bilobed ‘brain’ with different types of neurons and glia, and two longitudinal nerve cords connected by many transverse nerves [181,185]. Photo-, chemo- and rheoreceptors located at the front of the planarian’s body send signals to the brain, where they are processed, leading to behavioral responses [181,184]. Paired ‘eyes’ located on the planarian head allow the detection of light and shadow, and consist of two types of cells: pigmented optic cup cells, and photoreceptor neurons [189,190]. Due to the lack of respiratory and circulatory systems in planarians, oxygen is obtained and transported by diffusion. A centrally located pharynx is in charge of food intake and removal and is connected to a highly branched intestine, which circulates nutrients within the body. The excretory system (protonephridia) is responsible for the removal of waste products and osmoregulation [181]. Internal organs are surrounded by a mesenchymal tissue, parenchyma, consisting of adult pluripotent stem cells (neoblasts), which are essential for worms’ regeneration ability and comprise ~30% of the cells in the adult animal [185,191]. Planarians possess a set of muscle fibers, organized in longitudinal, diagonal, and circular orientations. The planarians body is covered with an epidermis; the ventral epidermis consists of a single layer of multiciliated cells (MCCs), and gland cells involved in the production and secretion of mucus, which is used by flatworms for protection, locomotion, catching food, and adhesion to substrates [181].

### 2.1. Advantages of the S. mediterranea as a Model Organism

Although planarians do not fully reflect the complexity of the human organism, many of the annotated *S. mediterranea’s* genes have known orthologs (or at least homologs) in the human genome, and researchers increasingly use *S. mediterranea* in studies aiming to better understand aspects related to human development and function involving certain cell types or tissues.

The maintenance of planarians is relatively easy and cheap, and does not require specialized equipment; only habitat conditions, such as temperature, darkness, feeding, and water culture, have to be provided, and many methodological guidelines have been published [e.g., [188,192,193]]. An important feature of using planarians as a model organism is the easy way to perform simple modifications of their gene expression. This can be achieved by knockdown/silencing genes of interest through RNA interference (RNAi) using double-stranded RNA (dsRNA). DsRNA can be administered to the worms by microinjection, by feeding them with dsRNA-containing bacteria, or with food mixed with free dsRNA [194,195,196]. The efficacy of gene silencing on the mRNA level can be evaluated using reverse transcription polymerase chain reaction (RT-PCR) or quantitative reverse transcription PCR (RT-qPCR), while the gene expression pattern of a silenced gene can be determined using whole-mount fluorescent in situ hybridization (FISH) and whole-mount in situ hybridization (WMISH). The phenotypic effect/s of gene silencing is typically observed within a week or two after implementing the RNAi procedure [188].

The genome of *S. mediterranea* has been well annotated, which makes this species more attractive than other planarians. The advances in single-cell RNA sequencing increased molecular knowledge about planarian stem cell differentiation, and have allowed for determining the transcriptomes for each cell type in *S. mediterranea,* and tracking the transcriptomic changes during the regeneration process [197,198,199,200]. The genomic and transcriptomic data are deposited in specialized databases and freely available to the research community [197,201,202,203,204].

### 2.2. S. mediterranea Model in the Context of Studying Cilia Biology

The potential of using *S. mediterranea* as a model organism to study evolutionarily conserved motile cilia was first described in 2009, and its value has been confirmed in later publications [188,192,205,206].

**Figure 2:**
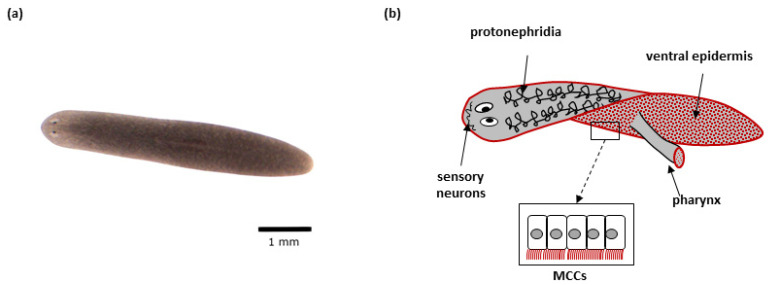
*Schmidtea mediterranea*: (**a**) Representative *S. mediterranea* individual–top view; (**b**) Schematic representation of a planarian. Tissues with cells featuring motile cilia are indicated. The red color indicates tissues/organs with multiciliated cells (MCCs).

Motile cilia are present in many planarian cell types (Figure 2). Multiple motile cilia (9 × 2 + 2) covering the apical side of MCCs (~80 per cell) in the planarian body epidermis beat in a synchronized way and are responsible for worms’ locomotion [188,207]. MCCs are also present in the epithelium that covers the feeding organ (pharynx) [188,207,208]. Specialized ciliated cells at the proximal end of protonephridia (so-called flame cells) play role in fluid ultrafiltration and circulation. Cilia with the same ultrastructure as motile ones are also found in the sensory neurons in planarians, although their ability to move is not clear [188,207]. Finally, sperm cells with flagella are present in the sexual strains of planarians [209,210]. In planarians, basal bodies are assembled de novo during terminal differentiation of ciliated cells from neoblast progenies, and never have the function of a centrosome [1,58,207]. While it is currently unclear how the flatworms generate multiple centrioles in cells that are initially centriole-free, RNAi experiments show that the known key factors of centriole duplication are crucial for their amplification [208].

Both ventral and pharyngeal epidermis are easily accessible and form cilia at high density and in known orientation [188]. The great advantage of using *S. mediterranea* as a model in motile cilia studies is that the effect of gene silencing on cilia function can be readily analyzed by recording the change in the speed of planarian locomotion. Importantly, defects that compromise the function and structure of the cilia are not detrimental to planarians, making them an ideal system for loss-of-function studies concerning cilia-related genes [192]. Under normal conditions, planarians move by the use of ciliated epithelium covering the ventral side of worms’ body (so-called gliding movement), while cilia-related gene silencing manifests in a so-called “inch-worming” movement that engages the muscles (waves of whole-body contraction and extension) [188,207]. This phenotype (inch-worming) is easily visible to even unaided human eyes; a stereoscope (with camera) makes it more precise and allows to record movies showing the movement of planarians, which can then be used to measure the distance traveled by worms (using ImageJ software) [192]. The motility impairment may be associated with edema, which results from the dysfunction of cilia in protonephridia [211]. The beating of cilia covering the lateral part of worms can be recorded using high-speed video camera microscopy (HSVM), and the cilia beating frequency, pattern, and synchrony can be analyzed in slow motion under a microscope. The number and length of cilia can be inspected using a fluorescence microscope after immunofluorescence staining (IF) with cilia-specific antibodies (acetylated alpha-tubulin, a marker of the axoneme). After RNAi, changes in the gene expression pattern of epidermal markers can be tracked using WMISH. In addition, the possibility to stimulate cell differentiation through worms’ fragmentation (cutting) that triggers the regeneration process allows for tracing changes in the gene expression during the differentiation of the neoblasts into ciliated cells. The effect of gene silencing on the ultrastructure of planarian cilia can be also examined using a transmission electron microscope (TEM). In addition, the flatworms bloat due to the inhibition of ciliary function in flame cells, which leads to defective osmoregulation and edema formation [207].

Planarians have been widely used as a model for studying signaling networks implicated in the maintenance of tissue homeostasis, regeneration, and polarity. A large number of studies were devoted to essential cellular pathways, including Wnt and Hedgehog signaling in establishing polarity [212], Akt signaling in tissue maintenance and regeneration [213], EGFR signaling in the regulation of excretory system [211]. Like in most bilaterally symmetric animals, canonical Wnt signal is transduced through frizzled receptor and with the help of disheveled stabilizes beta-catenin, which activates expression cascade controlling anterior/posterior axis during regeneration [212,214]. Wnt signals transduced through frizzled receptors to various non-canonical pathways (disheveled-dependent or Ca^2+^-dependent) control cell movement and planar cell polarity (apical positioning of the basal bodies of epithelial cells). Hedgehog signaling modulates Wnt/beta-catenin’s role in establishing the anterior/posterior axis; when Wnt signaling is low, heads develop, and when it is high, tails are formed [145,215]. The majority of these signaling networks have the ciliary context, linking various aspects of Hedgehog signaling, regeneration, and the biogenesis of cilia, e.g., [145,213,216,217].

### 2.3. S. mediterranea Model in the Context of Studying PCD and PCD-like Ciliopathies

RNAi-mediated silencing of a variety of genes in *S. mediterranea* has been used to explain/confirm the connection between the homologous genes, defects of the ciliary ultrastructure, and cilia dysfunction in other organisms. The explicit use of *S. mediterranea* as an animal model to elucidate the pathogenesis of motile ciliopathies (and in particular, the role of candidate PCD genes) includes relatively few studies, where the effect of gene silencing on motile cilia function has been examined using dsRNA-mediated knockdown of the planarian homologs of human candidate genes not previously linked to PCD. In many more *S. mediterranea* studies, the demonstrated phenotypic effects of motile cilia-related genes’ silencing strongly resemble those seen when PCD genes are mutated, but their involvement in the pathogenesis remains to be confirmed by finding deleterious variants in PCD patients.

Deleterious variants of ***CFAP298 (C21orf59)*** [81], ***CCDC151*** [109], and ***CFAP300 (C11orf70)*** **[63]** have been found in PCD patients. Knockdown of the planarian homologs of these genes has revealed the impaired locomotion phenotype in worms. The details of this phenotype have been explained using further assays. HSVM analysis of planarians with silenced *CFAP300 (C11orf70)* demonstrated changes in cilia motility pattern and lowered beat frequency, while TEM analysis of cilia in planarians with *CFAP298 (C21orf59)* and *CCDC151* knockdown revealed ODA assembly defects of dynein arms and loss of ODA, respectively. The effects of these three genes’ silencing are consistent with the observations in other animal models. *Ch. reinhardtii* and zebrafish mutants lacking *CCDC151* orthologues featured a loss of ODAs [111,218]; silencing of *CCDC151* in zebrafish and mice was shown to alter ODA assembly [109]. Knockdown of *CCDC298* in zebrafish and *Ch. reinhardtii*, and of *CFAP300* in *P*. *tetraurelia* and *Ch. reinhardtii* resulted in a complete lack of ODA and IDA [61,81]. All three genes are presently considered PCD genes, involved in the assembly of dynein arms (*CFAP298, CFAP300*), and proper functioning of the ODA docking complex (*CCD151*).

The role of FOXJ1 as the key transcription factor controlling motile cilia biogenesis has been reported in various FOXJ1-deficient model organisms, including mice [49], *X. laevis,* and zebrafish [50]. The *S. mediterranea* model has been used to demonstrate the conserved role of vertebrate *FOXJ1.* Among four ***FOXJ1*** homologs found in planarians, silencing of *FOXJ1-4* caused the absence of motile cilia, resulting in a characteristic inch-worming locomotion and edema formation [48]. The *FOXJ1* involvement in PCD pathogenesis in humans has been demonstrated several years later, when dominant pathogenic variants in *FOXJ1* were found in PCD patients with mild respiratory symptoms and hydrocephalus, caused by the severely reduced number of cilia per MCC due to defect in the apical docking of basal bodies [31].

Proteins essential to basal body assembly in *S. mediterranea* include orthologs of many conserved genes required for centriole assembly or function in humans. In planarians, depleting the ortholog of *OFD1* (among other proteins) results in the decreased locomotion of knocked-down animals, apparently due to the inhibition of basal body docking [58]. A similar ciliary phenotype has been recently demonstrated in PCD patients with the disease caused by nonsense mutations in the few last exons of the ***OFD1*** gene [55]. In humans, the truncation of the C-terminus of the protein causes PCD without severe neurological, skeletal, or renal symptoms characteristic for other *OFD1-*related syndromes associated with the loss of a larger part of the OFD1 protein cause syndromic diseases (e.g., oral-facial-digital syndrome type 1 or Joubert syndrome type 10). While the effect of the gene knockdown in planarians does not explain truncation size-dependent differences in human clinical phenotype, it corroborates the proposed mechanism for the ciliary phenotype in PCD patients, showing that apical docking of basal bodies in planarians and in humans employ, at least in part, the same molecular components.

***DAW1 (WDR69/ODA16)*** encodes a WD repeat protein, whose role as a dynein assembly factor has been shown in many model organisms. Depletion of DAW1 protein homologs results in ultrastructural defect characterized by the reduced number of ODAs in *Ch. reinhardtii* [219], zebrafish [220], and mouse [221]; *Ch. reinhardtii* studies have shown that DAW1 is involved in ODA transport through interaction with IFT46 protein [86,222,223]. The knockdown of *DAW1* homolog in *S. mediterranea* results in shortened epidermal cilia and decreased abundance of ciliated protonephridia [85]. The recent finding of deleterious *DAW1* variants in patients with disturbed laterality and respiratory symptoms has confirmed the predicted involvement of this gene in PCD pathogenesis, although only in patients whose cilia are characterized by subtle beating abnormalities [84].

Deleterious variants in two other genes, ***CFAP45/CCDC19/NESG1,*** and ***CFAP52/WDR16***, have been found in human individuals whose clinical presentation, with situs inversus and asthenozoospermia, but only mild respiratory symptoms, did not allow for classifying them as classical PCD cases [151]. Earlier studies in *Ch. reinhardtii* have localized these two proteins in the lumen of the B microtubule of the peripheral doublet [24]. The knockdown of the planarian homologs resulted in significant impairment of planarian locomotion in viscous but not in a normal medium; TEM of the silenced worms has shown normal ciliary ultrastructure, consistent with TEM cross-sections of *CFAP45*- and *CFAP52*-deficient respiratory cilia from CRISPR-Cas9 generated mice or from humans with mutated genes [151]. Therefore, planarian results confirm the uncertain status of *CFAP45* and *CFAP52* as PCD genes.

*IC2* and *LC1* are *S. mediterranea* homologs of human *DNAI2* and *DNAL1* genes, respectively, encoding integral components of ODA. Mutations in *DNAI2(IC2)* cause defects in ODA resulting in the reduction in ciliary beat frequency in *Ch. reinhardtii*, and are known to cause PCD in humans [90]. Mutations in ***DNAL1(LC1)*** disturb the proper function of ODA in *Ch. reinhardtii* [224], but the data supporting this gene’s role in human PCD are scarce [97,225]. The knockdown of either of these two genes in *S. mediterranea* severely decreases worms’ motility, due to the reduction in the ciliary beat frequency and coordination (metachronal synchrony). However, while TEM and IF reveal the loss of ODA in *IC2*-silenced planarians, no ODA defects are visible *in LC1-*silenced worms [91]. This is consistent with the still uncertain role of *DNAL1* in PCD pathogenesis in humans.

The ODA-docking complex is a microtubule-associated structure that targets ODA to its binding site on the axonemal microtubule [226]. In *Ch. reinhardtii* it contains three proteins, referred to as DC1, DC2, and DC3, of which DC1 and DC2 can assemble ODA in the absence of DC3 [227]. *Ch. reinhardtii* mutants with the loss of DC2 (a major subunit of the ODA-docking complex) have two flagella of normal length but show slow jerky swimming [228]. Two *DC2* homologs, *CCDC63* and *CCDC114,* function in ODA docking in vertebrates. Respiratory cilia in PCD patients with deleterious variants in ***CCDC114*** have normal length, but lack ODAs due to the defects in ODA docking to microtubules [229]. In mice, in which ***CCDC63*** (the testis-specific *DC2* homolog) is knocked out, spermatozoa flagella are shortened, but ODAs remain unaffected, probably due to the compensation by overexpression of *CCDC114* [230]. The knockdown of *DC2* orthologue in *S. mediterranea* impairs worms’ locomotion due to the low-frequency, uncoordinated ciliary beating caused by the inefficient ODA docking; in addition, cilia density and length are decreased [231]. The importance of these findings for PCD pathogenesis remains to be explored.

WDR92 is a highly conserved WD-repeat protein. Silencing of the planarian homolog of ***WDR92*** results in a phenotype similar to those observed when acknowledged PCD genes are knocked down. Peristaltic contractions instead of smooth gliding of the worms reduced and uncoordinated the ciliary beat; in TEM analysis, partial loss of dynein arms, incomplete closure of the B-microtubule, and lack of normal central pair complex are observed [232]. *WDR92* is required for the assembly of ODAs and IDAs in *D. melanogaster* and *Ch. reinhardtii* [233,234,235]. Based on these observations, *WDR92* has been proposed to act as a part of a cytoplasmic chaperone required for the proper folding and stability of key axonemal components. So far, no pathogenic variants have been found in human PCD patients.

***IFT88 (Tg737)*** encodes a component of the IFT complex; its mutations in *Ch. reinhardtii* results in a lack of flagella, while in mice they cause shortening of primary cilia, as well as kidney and liver defects [236]. The importance of *IFT88* in motile cilia biogenesis has been confirmed in the *S. mediterranea* model, where silencing of *IFT88* significantly reduced planarians motility and caused the complete absence of cilia on the ventral surface of knocked-down animals [91]. Defects in IFT are likely to affect motile cilia in humans. Defects in the *Tg737* gene in mice are very similar to those seen in humans with autosomal recessive polycystic kidney disease [237], but so far no pathogenic *IFT88* variants have been reported in PCD patients.

*Ch. reinhardtii* protein FAP163 is an intermediate dynein chain closely related to the FAP133 intermediate dynein chain that powers retrograde IFT required for the assembly of cilia. The functional role of FAP163 has been examined by the knockdown of the orthologous gene ***WD60 (FAP163)*** in *S. mediterranea* [238]. The silenced animals exhibited severely impaired movement (reduced velocity and inch-worming), resulting from a dramatic reduction in both the number and length of cilia. Cilia and ciliary stubs examined by TEM contained doublet microtubules and associated structures but had an enlarged diameter due to the presence of large quantities of amorphous electron-dense material located between the axonemal doublet microtubules and the ciliary membrane. These observations suggest that *WD60(FAP163*) is required for ciliary assembly. So far, no pathogenic variants have been found in human PCD patients.

An interesting application of RNAi-mediated gene silencing in *S. mediterranea* concerns the analysis of planarian protonephridia as a model of pathological features of human cystic kidney diseases (CKDs), in which fluid-filled cysts develop from nephric tubules due to defective flow sensing, cell proliferation, and differentiation. In contrast to mammalian kidneys that contain only immotile sensory cilia, the excretory system of planarians is equipped with motile cilia that drive fluid flow into and through the tubules [239,240]. Interestingly, structure and function comparisons revealed that the combination of ultrafiltration and flow-associated filtrate modification is remarkably conserved between the planarian excretory system (flame cells) and the vertebrate nephrons (podocytes) [67]. The genome of *S. mediterranea* contains many genes that cause cysts when their equivalents are mutated in humans. Silencing of planarian homologs of human ***DNAH1*** and ***LRRC50*** genes resulted in abnormal worms locomotion due to the loss of cilia beating; animals also developed edema and formed protonephridial cysts [67]. These results suggest that cilia-driven fluid flow is crucial for maintaining cell homeostasis in planarian protonephridia and establish planarians as a novel and experimentally accessible invertebrate model for the study of human kidney pathologies.

In the majority of the aforementioned studies, *S. mediterranea* was not the only organism used in the functional assessment of cilia-related genes, which are or can be considered candidate PCD genes. While the application of the planarian model in these studies may seem redundant, it can be seen that abundant work using *S. mediterranea* genes silencing has been performed to analyze the role of various proteins in the assembly, maintenance, and function of motile cilia, without immediate referring to PCD pathogenesis. These results are often used later, whenever deleterious variants in candidate PCD genes are found in human patients (see, e.g., the cases of *FOXJ1* or *DAW1*). On the other hand, when a new candidate PCD gene comes into focus based on genetic screening in humans, using the planarian model is perhaps the most efficient way to perform preliminary functional studies. When compared to the most popular single-cell organisms (*Ch. reinhardtii*, *P. tetraurelia*, *T. brucei*, *T. thermophila*), *S. mediterranea* offers an advantage of studying the epidermis that closely resembles human epithelium with MCCs, and compared to fish or mammals allows a much faster, easier and more affordable alternative modeling.

## 3. Perspectives

For many years, the use of the planarian model in the analysis of human candidate genes has been hampered by the lack of information on human-planarian orthologues. Recently, the rapid and extensive growth of the genomic, transcriptomic, phenotypic, and phylogenetic data generated by the planarian research community has alleviated this problem.

In this review, the human gene names or aliases are used to facilitate comparison with the part of the text describing human cilia and ciliopathies. However, it should be emphasized that to establish a uniform method for naming genes and proteins and for describing RNAi experiments in *S. mediterranea*, nomenclature guidelines have been developed [241], where the main rule is that the name of the gene is preceded by a ‘Smed’ prefix. In addition, to enhance cross-platform and cross-species searchability, the Planarian Anatomy Ontology (PLANA), an extendable relational framework of defined *S. mediterranea* anatomical terms has been recently developed [242].

The rapid growth of the amount of genomic and functional data on *S. mediterranea* prompted the development of integration tools that would enable the collection and use of these data by the scientific community. In response to these needs, the SmedGD database has been developed by the Sánchez Alvarado team [201,202]. The data deposited in SmedGD refer to the *S. mediterranea* genome and include predicted and annotated genes, protein homologies, gene expression patterns, and RNAi phenotypes, among others. SmedGD has been a stand-alone web resource for 15 years. Recently, the *S. mediterranea* genome assembly from SmedGD has been transferred to SIMRbase at the Stowers Institute for Medical Research (https://simrbase.stowers.org/ (accessed on 20 February 2023)).

Another high-quality *S. mediterranea* genome assembly can be found in the Planmine database (https://planmine.mpinat.mpg.de/planmine/begin.do (accessed on 20 February 2023)), developed in 2016 by the Rink team [199,203,243]. Originally, Planmine was based on the independently assembled transcriptomes from the Rink team and contributors from the planarian community. The updated database provides also genomic information, including a gene prediction set that assigns existing transcripts to defined genomic coordinates. In addition, Planmine uses recent datasets from the single-cell RNA-seq (e.g., from Digiworm resource and Planaria Single Cell Atlas), allowing for the expansion of the available gene expression information [197,198]. Both Digiworm and Planaria Single Cell Atlas refer to transcriptomes published by the Rink team, which makes these resources compatible with data in the Planmine database. Moreover, in contrast to other planarian databases, Planmine is also a resource of transcriptomes from other flatworm species. Planmine can be used to search for the planarian homologs of interesting transcripts and corresponding predicted genes. Planmine provides information about functional annotations (gene ontology), the best BLAST hits, expression patterns of homologs in planarian cell types, as well as phenotypes after RNAi of specified genes, among others.

Planosphere (https://planosphere.stowers.org/ (accessed on 20 February 2023)) is a new website dedicated to *S. mediterranea,* which contains a collection of data and tools from the Sánchez Alvarado laboratory [242]. One of the Planosphere tools is “gene search”, which can be used to search homologs in *S. mediterranea*, and to define experimentally determined cell/tissue-specific gene expression patterns. In addition to transcriptomic and genomic data, Planosphere provides information about predicted protein sequences. This website is linked to the data deposited in the Planmine database and refers to data (e.g., from RNAseq) published by other teams.

For researchers studying cilia or cilia-related diseases, it is important that RNAseq, together with other techniques, has allowed to characterize and reconstruct epidermal cell lineages, including the stages between neoblasts and fully differentiated epidermal cells [197,198,244,245,246]. Using Digiworm, researchers can check at which stage of epidermal lineage the gene of interest is expressed (it is possible for all cell/tissue types, e.g., protonephridia).

The available web resources, especially Planmine, Planosphere, and Digiworm, provide researchers with a powerful tool to design experiments using *S. mediterranea* as a model organism. They can be used to simply explore their contents to better understand planarian biology. Importantly, they allow the cross-searching of databases devoted to other organisms, using gene names, sequences, annotation terms, etc. (the details of such searches differ among planarian websites). This facilitates using the growing planarian knowledge in applications related to studies of human ciliopathies.

## Figures and Tables

**Table 1:** Genes involved in PCD pathogenesis.

Gene (Alias)	Role in Motile Cilia Biogenesis or Part of Axonemal Structure	Defects (Mode of Detection)	Confirmation from Selected Animal Models	Seminal References
Cytoplasmic proteins				
*CCNO* *MCIDAS*	Centriole amplification „	Sparse cilia (light microscope, TEM, IF against AcAT)	Xenopus Xenopus	[30] [29,30]
*FOXJ1*^1^; *autos. dominant* *RPGR*; *X-linked* *OFD1*; *X-linked* *GAS2L2*	BB stabiliz./orientation BB docking BB docking BB stabilization	BB mislocalized and more (TEM–except for GAS2L2; IF against BB elements)	Xenopus, Drerio, mouse, Smed Drerio Paramecium, Smed, mouse Mouse, Xenopus	[31,48,49,50] [51,52,53] [54,55,56,57,58] [59]
*DNAAF3 (c19orf51)* *CFAP300 (c11orf70)* *SPAG1 (DNAAF13)* *DNAAF1 (LRRC50)* *DNAAF5 (HEATR2)* *DNAAF6 (PIH1D3); X-linked* *DNAAF7 (ZMYND10)* *DNAAF11 (LRRC6)* *CFAP298 (c21orf59)* *DNAAF2 (KTU)* *DNAAF4 (DYX1C1)* *DAW1 (WDR69,ODA16)*	ODA/IDA preassembly „ „ „ „ „ „ „ „ Distal ODA preassembly “ “	Absent/shortened DA (TEM; IF against ODA or IDA elements)	Chlamy, Drerio Chlamy, Paramecium, Smed Drerio Chlamy, Tryp, Drerio, Smed Drosi, mouse Drerio, Drosi, mouse Drerio, Drosi, Xenopus, mouse Drosi, Drerio, mouse Drerio, Chlamy, Smed medaka, Chlamy, mouse Drerio, mouse Drerio, Smed, mouse, Chlamy	[60] [61,62,63] [64] [65,66,67,68] [69,70] [71,72,73,74] [75,76] [75,77,78,79,80] [81] [82] [83] [84,85,86]
*TTC12*	IDA assembly (in sperm IDA and ODA)	Some IDA types absent (TEM; IF against IDA elements, e.g., DNALI1)	Paramecium	[87]
**Elements of axonemal ultrastructure**				
*DNAI1* *DNAI2* *DNAH5* ^2^ *TXNDC3 (NME8; DNAI8)* *DNAL1* *DNAH11* ^3^ *DNAH9* ^3^	ODA “ “ “ “ Proximal ODA Distal ODA	Absent/shortened ODA (TEM–except for DNAH11; IF against ODA elements, e.g., DNAH5, DNAI2)	Chlamy Chlamy, Smed Chlamy Ciona Chlamy, Smed, Tryp Chlamy, mouse Chlamy, Paramecium, mouse	[88,89] [90,91,92] [93,94] [95,96] [91,97,98,99] [94,100,101] [94,102,103]
*CCDC114 (ODAD1)* *ARMC4 (ODAD2)* *TTC25 (ODAD4)* *CCDC151 (ODAD3)* *CCDC103* ^4^ *LRRC56 (DNAAF12)*	ODA targeting/docking „ „ „ Distal ODA targeting/docking “	Absent/shortened ODA (TEM; IF against ODA elements)	Chlamy Drerio, mouse Xenopus, mouse, Drerio Chlamy, Smed, Drerio, mouse Chlamy, Drerio Tryp	[104,105] [106] [107,108] [109,110,111] [112,113] [114]
*CFAP57(WDR65)*	IDA assembly	No TEM defect (IF)	Chlamy	[115,116]
*CCDC39 (CFAP59)* *CCDC40 (CFAP172)*	AR “	Mislocalized MTs, absent IDA (TEM; IF against AR elements or GAS8)	Drerio, mouse, dog Drerio, mouse	[117] [118]
*GAS8 (GAS11; DRC4)* *DRC1 (CCDC164)* *CCDC65 (DRC2; CFAP250)*	NDR complex „ „	MT mislocalized or no visible defect (IF against GAS8)	Chlamy, Tryp, Drerio, mouse Chlamy Chlamy, Drerio	[119,120,121,122] [123] [81,124,125]
*RSPH1* *RSPH4A* *RSPH9* *RSPH3* *DNAJB13 (RSPH16A)* *NME5 (RSPH23)*	RS head „ „ RS stalk „ RS neck	Central pair and MTs mislocalized (TEM except for DNAJB13; IF against RS’ elements)	mouse mouse Chlamy, Drerio, mouse Chlamy Chlamy, mouse Drerio, dog	[126,127,128] [129,130] [129] [131,132] [133,134,135] [136,137]
*HYDIN* *STK36 (FUSED)* *SPEF2* *CFAP74*	CP complex “ “ “	CP complex defects (no visible defect in TEM; IF against SPEF2, STK36; not for CFAP74)	Chlamy, mouse, Tryp Smed, Drerio, mouse mouse Chlamy	[138,139,140,141,142] [143,144,145] [139,146] [147,148]
*CFAP221 (PCDP1)*	“	No TEM defect	mouse, Chlamy	[148,149,150]

^1^ with hydrocephaly; ^2^ in sperm replaced by *DNAH8*; ^3^ in sperm replaced by *DNAH17*; ^4^ effect is variant-dependent. Abbreviations: AcaT—acetylated alpha-tubulin marker of microtubules; AR—axonemal ruler; BB—basal body; CP—central pair; IDA—inner dynein arm; IF—immunofluorescence; NDR—nexin-dynein regulatory; ODA—outer dynein arm; RS—radial spokes; TEM—transmission electron microscope; Xenopus—*Xenopus laevis*, Drerio—*Danio rerio*, Smed—*Schmidtea mediterranea*, Drosi—*Drosophila melanogaster*, Chlamy—*Chlamydomonas reinhardtii*, Ciona—*Ciona intestinalis*, Paramecium—*Paramecium tetraurelia*, Tryp—*Trypanosoma brucei.*

**Table 2:** Examples of genes with an uncertain role in PCD or involved in PCD-like diseases.

Gene (Alias)	Role in Motile Cilia Biogenesis or Part of Axonemal Structure	Relevance for PCD	Confirmation from Selected Animal Models	Seminal References
*CCDC19 (CFAP45)*	Inner lumen protein	Only mild respiratory symptoms	*Ch. reinhardtii*, *S. mediterranea,* mouse	[24,151]
*WDR16 (CFAP52)*	Inner lumen protein	Only mild respiratory symptoms	*Ch. reinhardtii*, *S. mediterranea*	[24,151,152]
*TP73*; *lissencephaly*	Ciliogenesis	Strong PCD candidate	mouse	[153,154]
*NEK10*	Centrosome; kinase	PCD candidate	medaka	[155,156]
*CCDC113 (CCDC96)*	NDR complex	PCD candidate	*T. thermophila*	[22]
*TEKT1*	Centrosome, BB, axoneme	PCD candidate	*D. rerio*	[157]
*CEP164*	Centriole; BB docking	PCD candidate	mouse	[158,159]
*CFAP206*	BB and axoneme	PCD candidate	*T. thermophila*, *Ch. reinhardtii*, *X*. *laevis*, mouse	[160,161,162]
*MNS1*	ODA docking	Laterality defect, male infertility	mouse	[163,164]
*BRWD1*	Axoneme	Male infertility	-	[165]
*CFAP43*	Axoneme	Male infertility	*X. laevis*, mouse	[166]
*CCDC11 (CFAP53)*	Ciliogenesis	Laterality defects	*X. laevis*	[167,168]

Abbreviations: BB, ODA, NDR are as in Table 1.

## Data Availability

Not applicable.
